# Simultaneous endovascular repair for abdominal aortic aneurysm and coronary artery bypass grafting in an octogenarian: A case report

**DOI:** 10.1016/j.ijscr.2019.11.036

**Published:** 2019-11-27

**Authors:** Tran Quyet Tien, Ho Tat Bang, Lam Thao Cuong, Nguyen Thai An

**Affiliations:** a Ho Chi Minh City University of Medicine and Pharmacy, Ho Chi Minh, Viet Nam; b Cho Ray Hospital, Ho Chi Minh, Viet Nam

**Keywords:** AAA, abdominal aortic aneurysm, CABG, coronary artery bypass grafting, CAD, coronary artery disease, CBP, cardiopulmonary bypass, CKMB, creatine kinase-muscle/brain, EVAR, endovascular aneurysm repair, GSV, great saphenous vein, LAD, left anterior descending, LCX, left circumﬂex, LIMA, left internal mammary artery, NSTEMI, non-ST-segment elevation myocardial infarction, RCA, right coronary artery, Abdominal aortic aneurysms, Coronary artery disease, Endovascular aneurysm repair, Coronary artery bypass grafting

## Abstract

**Introduction:**

Coronary artery disease (CAD) is commonly associated with abdominal aortic aneurysms (AAAs) in elderly patients. When severe CAD requiring coronary artery bypass grafting (CABG) is associated with an impending AAA in a high-risk patient, the options for the suitable timing of CABG and AAA repair strategy (one-stage or two-stage) are still being debated.

**Presentation of case:**

An 87-year-old man with non-ST-segment elevation myocardial infarction and a giant abdominal aortic aneurysm was transferred to our centre. Coronary angiography revealed triple-vessel coronary disease, and computed tomography confirmed a giant infrarenal fusiform abdominal aortic aneurysm 9 cm in maximal diameter. We simultaneously performed endovascular aneurysm repair prior to on-pump beating-heart coronary artery bypass grafting. The postoperative course was uneventful, and the patient was discharged on the 15th postoperative day.

**Conclusion:**

Combined endovascular aneurysm repair and on-pump beating heart coronary artery bypass grafting in a one-stage operation is a promising strategy to improve therapeutic efficiency in octogenarians. More studies are needed to compare the efficacy and safety of one-stage and two-stage operations to treat concomitant coronary artery disease and aortovascular pathology in the high-risk octogenarian patients.

## Highlights

• AAA is frequently associated with CAD in elderly patients.
• The optimal management of CAD associated with AAA in an octogenarian is a challenge.
• Combined CABG and EVAR is a promising way to improve the therapeutic efficiency.

## 1 Introduction

Coronary artery disease (CAD) is commonly associated with abdominal aortic aneurysms (AAA) in an elderly patient. The prevalence of AAA in patients with CAD has been reported to be approximately 8.4 % [1]. Conversely, approximately 65.3 % of AAA patients were reported to have concomitant CAD [2]. Some elderly patients present a combination of critical CAD, poor left ventricular function, and impending ruptured AAA. In this situation, finding the optimal treatment is a substantial challenge for clinicians. In the case of myocardial revascularisation prior to AAA repair, the risk of AAA rupture will significantly increase in the postoperative course [3,4]. On the other hand, if the AAA repair is carried out first, the sudden change in hemodynamic status will aggravate the existing myocardial ischaemia and have a detrimental effect on cardiac function [5]. Therefore, a one-stage surgery, simultaneous AAA repair and myocardial revascularisation was proposed for this situation [6,7]. Herein, we present a case of simultaneous coronary artery bypass grafting (CABG) and endovascular aneurysm repair (EVAR) in an octogenarian who had a non-ST-segment elevation myocardial infarction and large AAA. The patient provided written informed consent, this work was approved by institutional review board, and it has been reported in line with the SCARE criteria [8].

## 2 Presentation of case

An 87-year-old male patient was admitted to our centre from a local hospital with an initial diagnosis of non-ST-segment elevation myocardial infarction. The past medical history included controlled type II diabetes mellitus, hypertension, chronic obstructive pulmonary disease, and chronic coronary artery disease. On physical examination, blood pressure was 120/85 mmHg, heart rate of 102 beats per minute and peripheral capillary oxygen saturation of 89 %. Besides, a large pulsatile mass was palpated around the umbilical area.

**Fig. 1:**
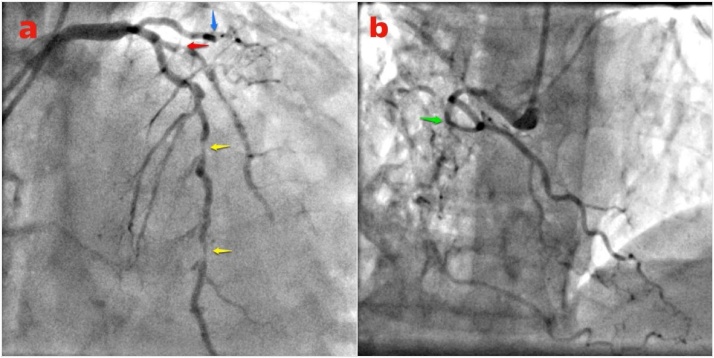
Coronary angiography revealed triple-vessel coronary disease. (a) 70–80 % stenosis of middle to distal left anterior descending artery (yellow arrows) and 90 % stenosis of diagonal artery 1 (red arrow). Total occlusion of the left circumﬂex artery indicated by the blue arrow. (b) Total occlusion of the right coronary artery (green arrow).

**Fig. 2:**
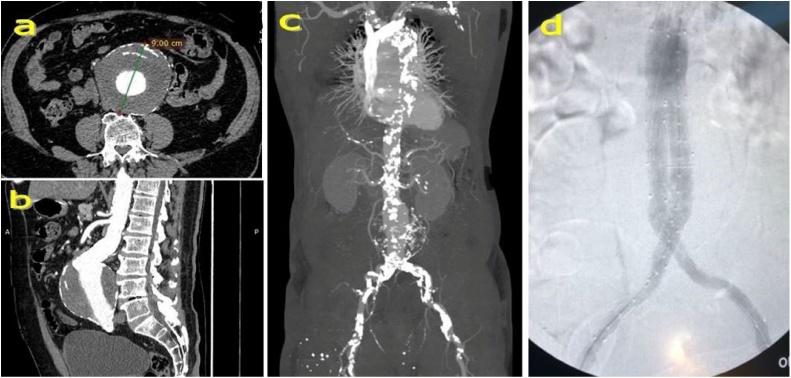
(a) Transverse and (b) sagittal plane view of the abdominal aortic aneurysm 9 cm in maximal diameter. (c) Arterial system of the body was in a severe level of atherosclerosis. (d) The stents successfully expanded without any leakage.

On the diagnosis workup, the electrocardiogram showed sinus rhythm with Q and ST-segment depression in V2 to V5 and in aVL. Echocardiography revealed severe left ventricular systolic dysfunction (EF 28 %) with global left ventricular hypokinesis. The laboratory data were abnormal with high levels of creatine kinase-muscle/brain and troponin I at 68.7 U/L and 500 ng/mL, respectively. Coronary angiography showed that the coronary artery system was right-dominant. The lesion was complex with triple-vessel coronary disease: (1) 70–80 % stenosis of middle to distal left anterior descending (LAD) artery and 90 % stenosis of diagonal artery 1; (2) total occlusion of the distal left circumﬂex (LCX) artery with collateral blood flow from LAD; and (3) total occlusion of the right coronary artery (RCA) with collateral flow from septal LAD and the ipsilateral with collateral flow from the RCA (Fig. 1). Apart from the complex lesions in the coronary arteries, computed tomography also revealed a giant infrarenal fusiform AAA 9 cm in maximal diameter (Fig. 2 a–b). Severe atherosclerosis of the entire arterial system was also noted (Fig. 2c).

According to the SYNTAX score (46.5 points) calculated as described previously [9], these lesions were not suitable for percutaneous coronary intervention. Thus, CABG was the only treatment option. For the huge AAA, the indication for AAA repair was clear to avoid the rupture of the aneurysm. The patient’s condition was critical because of the high likelihood of death from aortic aneurysm rupture and myocardial infarction. The risk of in-hospital mortality was 35.25 % (EuroSCORE II). Thus, with urgent multidisciplinary meeting (including cardiac surgeons, vascular surgeons, cardiologists, anaesthesiologists, and intensivists), we decided to simultaneously perform AAA repair and CABG. For AAA repair, we chose endovascular repair (EVAR) instead of the more invasive open repair that has a high risk of mortality.

Under general endotracheal anaesthesia, EVAR was first carried out followed by CABG. Preoperative planning and sizing for EVAR was done on the basis of a three-dimension analysis using OsiriX software. Arterial access was achieved by exposing bilateral femoral arteries. The Endurant II AAA Stent Graft System (including main body 28-14-103 mm, 16-13-124 mm stent for left common iliac artery, 16-10-156 mm stent for right common iliac artery) was successfully expanded without any leakage (Fig. 2d). The total time for EVAR was 65 min. Immediately after terminating EVAR, on-pump beating-heart CABG was carried out under full median sternotomy and the support of a cardiopulmonary bypass (CBP). CBP was set through the right atrium and ascending aorta. The arterial coronary revascularisation was fulfilled using the great saphenous vein (GSV) and the left internal mammary artery (LIMA). The connections were established including (1) LIMA – LAD; (2) brachiocephalic artery - obtuse marginal artery - posterior descending artery (by GSV). Total time for completing coronary revascularisation and cardiopulmonary bypass was 65 and 90 min, respectively. The postoperative hemodynamics was stable, and the patient was extubated on the second postoperative day. The postoperative course was uneventful, and the patient was discharged on the 15th postoperative day.

## 3 Discussion

In the treatment of patients with large or symptomatic AAA associated with critical CAD, although combined AAA repair and CABG in a one-stage operation has been sporadically reported, the optimal strategy and clinical outcomes are still debated, especially in elderly patients with multiple comorbidities and poor left ventricular function. A simultaneous surgery has some beneficial aspects, such as diminishing the risk of aneurysm rupture. Indeed, Paty et al. reported that 23 % of their patients had AAA rupture in the period after CABG [4]. Blackbourne et al. also presented that 33 % of their patients died as a result of ruptured AAA 16–29 days after CABG [3]. Furthermore, the one-stage operation can avoid repeated anaesthesia, shorten the hospital stay, and decrease the hospital cost [6,10]. However, performing two procedures under the same anaesthesia may be too invasive, especially in a elderly patient with multiple comorbidities. Some detrimental results were reported in some literature such as the high rate of mortality and morbidity in the postoperative course of combined surgery, this risk was especially higher in patients with low ejection fraction [11]. Therefore, the option for either a one-stage or two-stage procedure should be individualised and considered depending on the specific condition of each patient, on the experience of the surgical team, and on the actual conditions of each centre.

In our case, aside from the serious clinical condition, non-ST-segment elevation myocardial infarction was confirmed and required CABG. The giant AAA 9 cm in diameter was impending rupture and needed to be prevented. We performed simultaneously EVAR and CABG because of the advantages of EVAR in high-risk patients [12]. EVAR is more feasible, safer, and less invasive than open repair. It is performed via bilateral femoral artery exposure, and a minimal incision can reduce the risk of bleeding. The hemodynamic change is negligible owing to the absence of aortic clamping. Thus, patients can tolerate the procedure [13]. For reducing the invasiveness of CABG, we utilized the on-pump beating-heart technique, which has recently been proposed for high-risk patients, especially for elderly patients who has poor left ventricular function [14,15]. This technique may have the potential to acquire the advantages of both on-pump and off-pump procedures while avoiding their disadvantages [16].

To conclude, although there were a lot of reports of octogenarian patients who have been successfully treated by simultaneous CABG and EVAR, the ideal strategy for this subgroup of patients is still debated and is a challenge for clinicians. Combined on-pump beating-heart CABG and EVAR is a promising way to improve the therapeutic efficiency. Nevertheless, further studies are needed to compare the efficacy and safety of one-stage and two-stage operations.

## Sources of funding

No funding was received for this case report.

## Ethical approval

Our Institute’s (Cardiovascular Center) representative was fully aware of this submission and this scientific activity including writing manuscript was approved by the Ethic Committee of Cho Ray hospital, where the patients were operated.

## Consent

Written informed consent was obtained from the patient for publication of this report and accompanying images. A copy of the written consent is available for review by the Editor-in-Chief of this journal on request.

## Author contribution

Tran Quyet Tien and Nguyen Thai An: Surgeon, the conception and design of the study, revising it critically for important intellectual content, final approval of the version to be submitted.

Lam Thao Cuong and Ho Tat Bang: Acquisition, analysis and interpretation of data, drafting the article, final approval of the version to be submitted.

## Registration of research studies

This work does not apply as it is a case report of a patient who has given written consent and has been de-identified. It is therefore not prospective research involving human participant

## Guarantor

Tien Quyet Tran, M.D., PhD.

## Authorship

All authors attest that they meet the current ICMJE criteria for Authorship.

## Provenance and peer review

Not commissioned, externally peer-reviewed

## Declaration of Competing Interest

The authors declare that they have no competing interests.
